# The Gut Microbiota-Brain Axis: A New Frontier on Neuropsychiatric Disorders

**DOI:** 10.3389/fpsyt.2022.872594

**Published:** 2022-06-01

**Authors:** Sarha A. L. Queiroz, Alyne M. M. Ton, Thiago M. C. Pereira, Bianca P. Campagnaro, Larissa Martinelli, Aitor Picos, Manuel Campos-Toimil, Elisardo C. Vasquez

**Affiliations:** ^1^Laboratory of Translational Physiology and Pharmacology, Pharmaceutical Sciences Graduate Program, Vila Velha University, Vila Velha, Brazil; ^2^Federal Institute of Education, Science and Technology (IFES), Vila Velha, Brazil; ^3^Physiology and Pharmacology of Chronic Diseases (FIFAEC), Center for Research in Molecular Medicine and Chronic Diseases (CIMUS), University of Santiago de Compostela, Santiago de Compostela, Spain

**Keywords:** Alzheimer's disease, microbiota-gut-brain axis, dysbiosis, probiotic, neuropsychiatric symptoms

## Abstract

Alzheimer's disease (AD) is a progressive and incurable neurodegenerative disorder of integrative areas of the brain, characterized by cognitive decline and disability resulting in negative impacts on the family of the patients and the health care services worldwide. AD involves oxidative stress, neuroinflammation and accelerated apoptosis, accompanied by deposition of amyloid-β peptide plaques and tau protein-based neurofibrillary tangles in the central nervous system. Among the multiple factors that contribute to the onset and evolution of this disease, aging stands out. That is why the prevalence of this disease has increased due to the constant increase in life expectancy. In the hope of finding new, more effective methods to slow the progression of this disease, over the last two decades, researchers have promoted “omics”-based approaches that include the gut microbiota and their reciprocal interactions with different targets in the body. This scientific advance has also led to a better understanding of brain compartments and the mechanisms that affect the integrity of the blood-brain barrier. This review aims to discuss recent advances related to the gut-brain-microbiota axis in AD. Furthermore, considering that AD involves psychiatric symptoms, this review also focuses on the psychiatric factors that interact with this axis (an issue that has not yet been sufficiently addressed in the literature).

## Introduction

With the aging of the population, as a result of the change in the age pyramid, neurodegenerative diseases are increasing their percentage as a cause of death and disability, besides representing a large and growing economic impact on health care (1, 2). Among neurodegenerative diseases, Alzheimer's disease (AD) is the most important, since it corresponds to 60% of dementias, being the most prevalent etiology worldwide (3). The costs related to the treatment and medical care of people with AD are considerably high and dementia is one of the most expensive conditions for society (4). Thus, AD stands out in the development of public policies to increase survival, reduce the socioeconomic impact, as well as improve the quality of life of people diagnosed with AD and the caregivers and family members (3).

AD affects the various cognitive domains of an individual, such as memory, executive functions, language, behavioral habits, as well as visuospatial skills and attention (5). Symptoms are explained by progressive synaptic loss, cholinergic dysfunction, and neuronal death observed in brain regions responsible for cognitive functions, especially in the cerebral cortex, hippocampus, and striatum (6–8). Such neuropathological findings appear before the manifestation of cognitive deterioration and neuropsychic dysfunctions (9).

In this context, intestinal dysbiosis has been strongly associated with the etiopathogenic mechanisms of cognitive and neuropsychiatric dysfunctions of AD and may be closely related to the promotion of neuroinflammation, oxidative stress, hyperphosphorylation of tau protein, and aggregation of amyloid-β protein (Aβ) (10, 11).

Aging, a main risk factor for AD, involves a process of changes in the gastrointestinal system: hypochlorhydria, difficulties in transit and intestinal motility, degenerative changes in enteric nerve cells, reduced absorption capacity as well as changes in the quality and quantity of the intestinal microbiota (12–14). Therefore, scientific evidence indicates the importance of understanding the underlying mechanisms involved in the brain microbiota-gut axis as a promising pathway for new therapeutic strategies for the prevention and treatment of AD.

Gut-brain cross-talking is crucial in the regulation of the central nervous system (CNS) and the gastrointestinal system, and several studies have characterized its importance in the homeostatic health-disease process (15–18). In recent years, there has been a growing interest in the characterization of the gut microbiota as a main regulator of this bidirectional communication that impacts the neurophysiological bases of psychiatric and neurodegenerative disorders (19–21). Thus, the development of technologies for the study of microbiota-brain communication has gained prominence, as well as the investigation of new therapies for neuropsychiatric symptoms through intestinal modulation. This review aims to update knowledge about the role of the gut-brain-microbiota axis in the development and prevention of neuropsychiatric symptoms in Alzheimer's disease (AD).

## Gut Microbiota, Aging and Alzheimer's Disease

The gut microbiota comprises a range of microorganisms of around 1,000–5,000 different non-redundant species. The vast majority correspond to bacteria belonging to Phyla Firmicutes, Bacteroidetes, Actinobacteria, Proteobacteria, Fusobacteria and Verrucomicrobia, and the existence of 150 times more genes than in the human genome has been demonstrated (22, 23). It consists of more than 100 trillion microbial cells that interfere not only in intestinal and absorptive functions, but also in a wide network of neuronal, mental, immunological, endocrine, and metabolic actions (24).

The intestinal microbiota plays important functions in the body, including the maintenance of a mucous barrier of enterocytes responsible for a complex system of defense against pathogens in the host, the control over intestinal permeability and the regulation of absorption, immunomodulation, and anti-inflammatory mechanisms (25). In addition, it is responsible for metabolic pathways of production of vitamins, hormones, amino acids, and the biotransformation of short-chain fatty acids (26).

**Figure 1:**
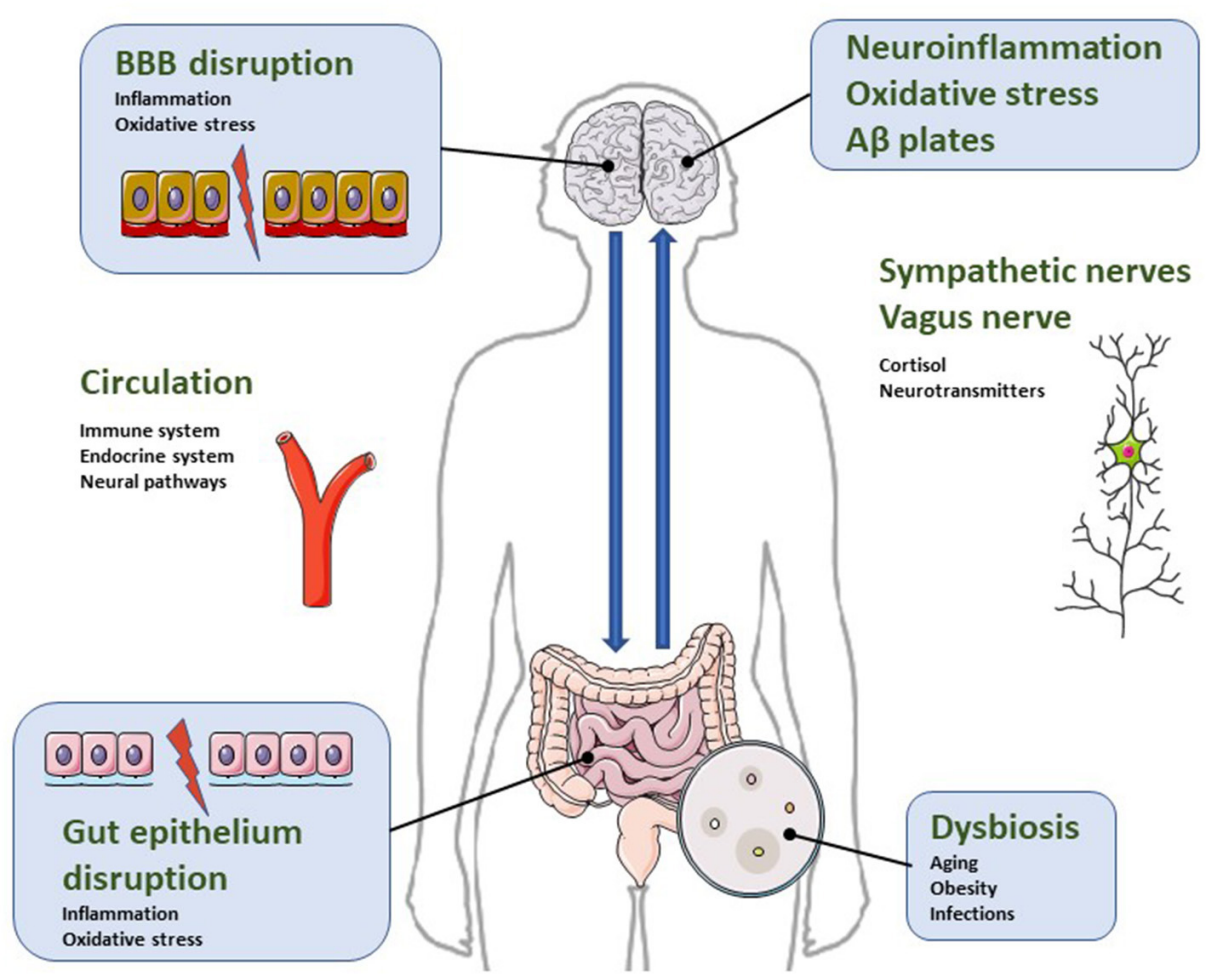
Illustrative diagram regarding the bidirectional relationship of the microbiota-gut-brain axis in the pathophysiology of neuroinflammatory parameters characteristic of AD. Dysbiosis is a condition characterized by increased intestinal permeability due to gut epithelial disruption. It impacts microglial and astrocytic activation and causes blood brain barrier disruption. Consequently, dysbiosis activates neuroinflammatory pathways, oxidative stress and neuronal dysregulation, leading to apoptosis and accumulation of β-amyloid protein. For more details, see text.

Gut-brain communication is based on signals generated in the gut microbiota that send and receive information from distant organs. This axis of information includes neural pathways through autonomic nervous system with branches of the vagus nerve, endocrine transmission through hormones (mainly the hypothalamus-pituitary-adrenal system) and immunological propagation through chemokines and cytokines (16, 27, 28) (Figure 1).

**Figure 2:**
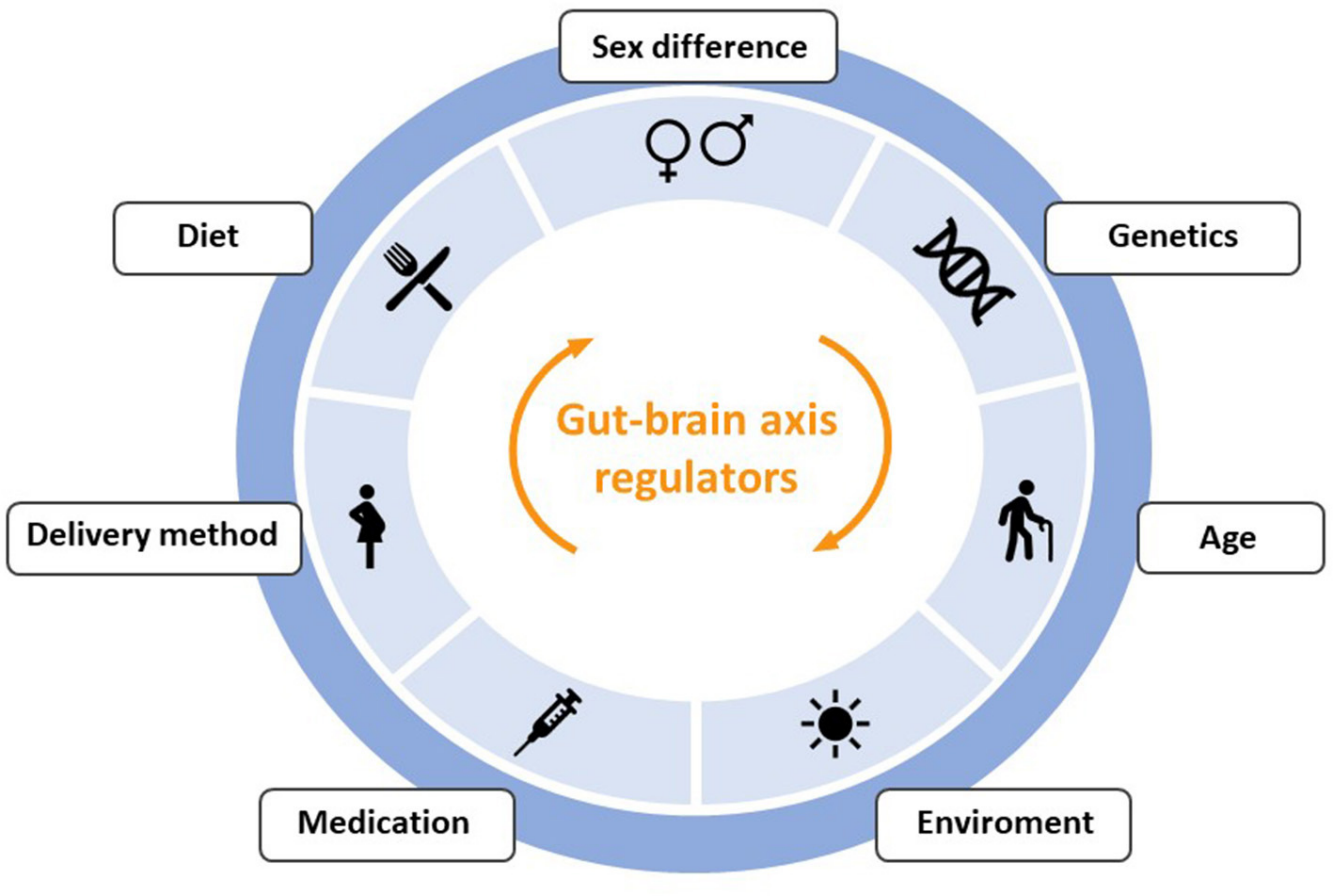
Outline of the main factors that can produce alterations in the communication between the intestine and the brain through the gut-brain-axis.

This symbiotic community of non-pathogenic microorganisms has wide variability and changes due to exposure to intrinsic and extrinsic factors throughout life, such as use of antibiotics, sedentary lifestyle, infections, diet, aging, cesarean sections, genetics, chronic stress and lack of breastfeeding (29–32). Some of the factors that regulate communication through the gut-brain axis are represented in Figure 2, although their review is beyond the scope of this article.

The imbalance in the composition of the intestinal microbiota is called intestinal dysbiosis and triggers a cascade of dysfunctional processes, including neuropsychiatric symptoms of neurodegenerative diseases such as AD (33–36) (Figure 1).

With age, microbiota suffers a reduction in the amount of Lactobacillus, Bifidobacteria, Bacteroidetes, Firmicutes, Lachnospiraceae, Ruminococcaceae, Coprococcus, Roseburia and Faecalibacterium, and an increase in the proportion of Odoribacter and Butyricimonas (37–39). Such changes are associated with gastrointestinal disorders that favor increased intestinal permeability, dysbiosis, and the development of a neuroinflammatory cascade (Figure 1) (40, 41).

## Microbiota and Neurotransmitters

Neurotransmitters play an important role in the etiopathogenic mechanisms involved in the neurodegeneration of Alzheimer's disease since they are responsible for the satellite transmission and interneuron communication (Table 1) (42). According to this, the main agents approved for the treatment of AD are acetylcholinesterase inhibitors (donepezil, tacrine, galantamine and rivastigmine), which prevent the decrease in acetylcholine, a neurotransmitter related to cognitive functions such as learning and memory, in the synaptic cleft (43).

**Table 1:** Scientific evidence of the association of bacteria, neurotransmitters, and neuropsychiatric effects.

**Neuromodulators**	**Bacteria**	**Effects**	**References**
Acetylcholine	Bacillus; *Bifidobacterium bifidum Lactobacillus plantarum*	Neurotransmitter related to cognitive functions such as learning, memory and executive abilities of daily living.	(46)
Gamma-aminobutyric acid (GABA)	Lactobacillus and Bifidobacterium	Main inhibitory neurotransmitter of the CNS, responsible for information processing, mood besides being involved with sleep and modulation of muscle contractions.	(48)
Glutamate	*Campylobacter jejuni*	The main excitatory neurotransmitter of the CNS, being associated with the ability to find alternative ways to solve problems, increased mental timing and information processing efficiency.	(55, 56)
Serotonin	Streptococcus, Escherichia, Enterococcus, Lactococcus and Lactobacillus	Linked to the regulation of emotions, mood, appetite, sleep and cognitive functions.	(61, 62)
Brain-derived neurotrophic factor (BNDF)	*Lactobacillus plantarum* IS-10506	Main neurotrophin present in the CNS, involved in the maintenance of neuronal homeotase, allows adequate growth and differentiation of neural cells and synapses.	(69)
Dopamine	Escherichia, Bacillus, *Staphylococcus aureus* and *Serratia marcescens*	Dopaminergic pathways are associated with neuropsychiatric effects on mood control, emotion, behavior, as well as in command of movements.	(70, 73, 75)

Gut microbiota participates in the production and release of neurotransmitters through enteroendocrine cells, which consequently interfere with CNS functions (44). Strains of Lactobacillus have been shown to be capable of producing acetylcholine (45). Furthermore, in an animal model of AD, the administration of *Bifidobacterium bifidum* and *Lactobacillus plantarum* for 8 weeks, associated with physical training, relieved amyloid-β protein neurotoxicity and improved spatial learning by an acetylcholine-mediated mechanism (46).

The main CNS inhibitory neurotransmitter, γ-aminobutyric acid (GABA), important for maintaining neuronal homeostasis, is produced by strains of Lactobacillus and Bifidobacterium (47, 48). Enteric dysbiosis reduces GABA in the gastrointestinal tract and brain, unbalancing the GABA/glutamate ratio, increasing the excitotoxic potential and leading to AD progression (49). In fact, GABA levels are reduced in the cortex of AD patients (50). Also, in experiments with diet-induced obese and metabolically dysfunctional mice, GABA production by lactobacilli has been shown to reduce metabolic and depressive behavior disturbances (51).

Glutamate, the main excitatory neurotransmitter in the CNS, is also a key part of balanced synaptic transmission and neuronal plasticity, since it acts directly through N-methyl-D-aspartate (NMDA) receptors in the acquisition of new knowledge, as well as storage capacity (52, 53). An overactivation of glutamate may be involved in neurotoxicity, leading to neuronal damage/death, which justifies the introduction of memantine (NMDA antagonist) in the pharmacotherapy of AD. Research in humans has associated glutamate metabolism by the gut microbiota with neurocognitive functions, such as the ability to find alternative ways to solve problems, increased mental timing, and efficiency in information processing (54). Finally, *Campylobacter jejuni* was associated with a stimulus to glutamate synthesis through direct stimulation of the enzyme γ-glutamyltranspeptidase (GGT) (55, 56).

Other authors have shown an association between intestinal diseases and neuropsychiatric disorders. One study identified reduced anxiety-like behavior associated with the absence of gut microbiota in mice, related to lower expression of NMDA receptor messenger RNA in the amygdala (57). Also, colitis induction in mice generated anxious behaviors and cognitive loss associated with the reduction of intestinal bacteria due to acute local inflammation, mainly lactobacilli and segmented filamentous bacteria. These effects were prevented by administration of probiotics containing *Lactobacillus rhamnosus* R0011 and *Lactobacillus helveticus* R0052 (58).

The serotonergic system is also associated with brain dysfunctions such as mood swings, cognition, and the circadian cycle (59, 60). Serotonin is a product of tryptophan metabolism and about 90% of its synthesis occurs through bacteria of the gastrointestinal tract, such as Streptococcus, Escherichia, Enterococcus, Lactococcus and Lactobacillus (61, 62). Tryptophan concentrations are increased in the plasma of germ-free male animals and anxiety normalized after restoration of the gut microbiota, reinforcing the importance of bidirectional communication of the gut-brain axis in serotonergic neurotransmission (63).

Eubiosis (a healthy and balanced bacterial ecosystem) was also related to the regulation of brain-derived neurotrophic factor (BNDF) levels in brain regions associated with memory, learning, cognition, and visuospatial abilities (64, 65). BDNF mediates important beneficial effects of exercise, including various neuronal processes of development and neuroplasticity (66), and a reduction in its expression is considered a reliable marker for AD, as it can exacerbate pathophysiological tau protein and β-amyloid deposition (67). BDNF has been shown to decrease Aβ production by enhancing α-secretase processing of amyloid precursor protein (APP) (68). Upregulation of brain BDNF expression was shown in rats after probiotic treatment containing *Lactobacillus plantarum* IS-10506, suggesting its prophylactic value against AD (69).

In addition, studies increasingly demonstrate the interaction of the dopaminergic system with the neuropsychiatric symptoms of AD (70). Neuronal death and age-dependent dopaminergic degeneration were observed exclusively in the ventral tegmental area in pre-plaque stages in a mouse model of AD (71). Dopaminergic neurons are located mainly in the ventral tegmental area, which is responsible for the motivation and reward system, and in the substantia nigra pars compact, mainly involved in movement control. Dopamine may be directly related to neuropsychiatric symptoms common in AD, such as anhedonia and apathy (72). Also, the cholinergic deficit in AD was transiently restored by the administration of a dopaminergic receptor agonist under transcranial magnetic stimulation (73, 74). Finally, the *in vitro* production of dopamine by several bacteria, including *Bacillus cereus, Bacillus mycoides, Bacillus subtilis, Escherichia coli, Serratia marcescens*, and *Staphylococcus aureus*, has been reported (75, 76).

This experimental and clinical evidence suggests the relevance of the gut microbiota not only for host intestinal health, but also for neurocognitive protection and improvement of clinical outcomes.

## Microbiota and Neuroinflammation in AD

Neuroinflammation has been reported to play an important role in the pathophysiological mechanisms of AD (77, 78). It may have a peripheral origin, since increased peripheral cytokine production may lead to endogenous cerebral cytokine production (79). Thus, intestinal inflammatory processes can affect brain function, and have been postulated as a prominent factor in the progression of neurodegenerative diseases (80). Symptoms associated with cognitive and functional impairment of Alzheimer's, such as apathy (81) or agitation (82) have been related to an increase in peripheral proinflammatory mediators. In fact, there is considerable experimental evidence indicating that systemic inflammation causes disruptive effects on the blood brain barrier BBB, facilitating the entry of inflammatory factors into the brain, a process implicated in the pathogenesis of neurodegeneration (83, 84) (Figure 1).

The disruptive processes of the BBB that allow the entry of inflammatory factors into the CNS have been related to altered states of the intestinal microbiota. In 2014, a study showed that germ-free mice showed increased permeability of their BBB, compared to mice with normal gut flora, and a lower permeability of the barrier could be restored by a recolonization of the normal microbiota (85). In fact, eubiosis helps keep intestinal intercellular junctions intact, favoring a correct @ control of intestinal permeability, which prevents the entry of proinflammatory substances into the bloodstream (86). However, dysbiosis increases permeability, giving rise to a dysfunctional intestinal barrier and culminating in an inflammatory process that compromises the integrity of the BBB (87), and, therefore, facilitates the onset of neurodegenerative processes.

Other studies have also suggested a relationship between alterations in the intestinal microbiota and neuroinflammation. Inflammatory neurodegeneration could be triggered by the activation of the pro-inflammatory nuclear factor κB (NF-kB) by lipopolysaccharides produced by Bacteroidetes species (88). Also, the induction of cerebral amyloidosis in patients with AD has been related to an inflammatory process due to an imbalance between proinflammatory bacteria (Escherichia and Shigella) to the detriment of beneficial species, mainly *Escherichia rectale*. Likewise, the reduction of proinflammatory cytokines has been related to the presence of anti-inflammatory intestinal bacteria in patients with dementia and amyloidosis (89).

Kim et al. (90) showed that the gut microbiota of mice with amyloid disease and neurofibrillary tangles had a loss of epithelial barrier integrity in addition to gut inflammation. Interestingly, transplantation of fecal microbiota from healthy wild-type mice to diseased mice reduced the formation of β-amyloid plaques and neurofibrillary tangles, glial reactivity, and cognitive impairment and also reversed abnormalities in colonic gene expression related to the activity of intestinal macrophages and circulating inflammatory monocytes in the blood (90).

Using transgenic animals, other researchers have shown that fecal microbiota transplantation reduces amyloid plaques, inflammatory markers, and improves neuroplasticity and cognitive aspects (91). Therefore, modulation of the gut microbiota by probiotics and fecal microbiota transplantation interventions is relevant to protect the host's cognitive behavior, an effect in which the reduction of neuroinflammation is involved (92).

For a recent review on the role of the microbiota in neuroinflammation and synaptic dysfunction in AD see, e.g., Bairamian et al. (41).

## Microbiota and Oxidative Stress in AD

Oxidative stress leads to increased inflammation through mitochondrial destruction and activation of astrocytes by reactive oxygen species (ROS) (93). According to the oxidative stress theory, neuronal death in AD is due to an imbalance between ROS production and clearance. Thus, the excessive concentration of ROS acts on the membranes of neuronal cells, causing a change in permeability and, consequently, an alteration in cell communication and neuronal signal transduction through a deregulation of calcium influx (94, 95).

In addition to this loss of neuronal synapses, oxidative stress contributes to the pathogenesis of AD through participation in the process of abnormal proteolytic cleavage of APP with production and deposition of the β-amyloid substance, formation of senile plaques, and hyperphosphorylation of tau protein (96, 97). In this way, oxidative stress contributes not only to the onset of AD, but also to the progression and severity of the disease (98). Recently, the importance of oxidative stress in the pathogenesis, diagnosis and monitoring of various neurodegenerative diseases, including AD, has been reviewed (99, 100).

Thus, the regulation of oxidative stress could play an important role in the control of these diseases. In this sense, the maintenance of eubiosis can play a prominent role. In fact, intestinal microbiota plays an important role in regulating oxidative stress. Gastrointestinal bacteria such as lactobacilli and bifidobacteria can convert nitrate and nitrite into nitric oxide (NO) (101). Moreover, species such as streptomycetes and bacilli synthesize NO from L-arginine through the enzyme NO synthase (NOS) (102). NO is essential in the transmission of information from the noradrenergic enteric nervous system, besides being excreted by the glutamatergic stimulus *via* activation of NMDA receptors. Although it has an essential role in neuroprotection, exacerbated NO levels can also cause oxidative stress, culminating in apoptotic cell damage and axonal degeneration (103). In this sense, intestinal eubiosis is important, since its ability to stimulate microglia leads to increased production of inducible NOS (iNOS), increasing NO production and helping to maintain immune balance (104, 105).

Recently, we have demonstrated that synbiotic kefir supplementation in elderly AD patients improves cognitive dysfunction on specific tests, with benefits related to memory, language, executive functions, visuospatial function, conceptualization, and abstraction skills. This outcome was observed with an increased NO and a reduction in protein oxidation and ROS levels (106).

## Microbiota and Hyperactivation of the Hypothalamic-Pituitary- Adrenal Axis by Stress

Chronic stress is a physiological adaptive response of the organism to external events that can cause some harm to the individual and a risk factor for the development of neurodegenerative diseases, including AD. In fact, a neuroadaptive response to sustained and chronic stress causes immunological and neuroendocrine imbalance, increases inflammatory activity, and generates behavioral and psychological changes (107–109).

The hypothalamic-pituitary-adrenal (HPA) axis regulates the response to acute and chronic stressors, such as sedentary lifestyle, altered circadian rhythm, alcohol abuse, and exposure to noisy environments (110–112). The HPA axis responds to stress with the production of corticotrophin-releasing hormone (CRH) by the hypothalamus. CRH stimulates the pituitary gland to produce adrenocorticotrophic hormone (ACTH), which, in turn, acts on the adrenal glands to release cortisol (113). Persistent overactivation of the HPA axis leads to neuroinflammation, neuroendocrine dysfunction, hippocampal atrophy, and consequently cognitive deficits and neuropsychiatric symptoms (114, 115).

While chronically elevated cortisol levels negatively affect brain function, activation of the HPA axis also modifies the composition of the gut microbiota, causing dysbiosis (116). Treatment with prebiotics containing fructooligosaccharides and galactooligosaccharides had antidepressant and anxiolytic effects associated with reduced levels of corticosteroids caused by stress (117). Accordingly, recent studies have shown that a treatment with fructooligosaccharides could ameliorate the cognitive impairment and neuropathology change, as well as alleviate Aβ accumulation in the brain of the APP/PS1 double transgenic mice model of AD (118, 119). Similarly, galactooligosaccharides have been reported to reverse cognitive behavioral impairment in APP/PS1 mice, as well as reduce their levels of depression (120). In addition, the intake of lactobacilli was related to the improvement of cognitive parameters and the decrease in serum corticosterone levels (121).

A probiotic formulation consisting of *Bifidobacterium longum* 1714 given to 22 healthy male volunteers was able to mitigate increases in cortisol levels and subjective anxiety in response to an acute stressor. In addition, there was also an improvement in hippocampal-dependent visuospatial memory performance and changes in brain activity, as assessed by electroencephalography (122).

In another clinical study, the consumption of a probiotic formulation composed of *Lactobacillus helveticus* R0052 and *Bifidobacterium longum* R0175 in combination mitigated psychological distress without showing adverse effects, demonstrating the role of intestinal flora in stress, anxiety and depression (123).

Finally, germ-free mice exhibited higher levels of ACTH and corticosterone compared to the control group in response to restraint stress. This effect was fully reversed after reconstitution with *Bifidobacterium infantis* (124). Thus, the interaction of the intestinal microbiota was verified as an important factor in the stress response of the HPA axis.

## Conclusion and Expected Forthcoming Advances

The gut microbiota is associated with functions that extend to the local gastrointestinal context, but are related to the modulation of important endocrine, metabolic, immunological, and neural pathways in the body. The existence of the microbiota-brain-gut axis demonstrates how crucial the maintenance of eubiosis is for brain homeostasis. In the context of AD, the intestinal microbiota plays a key role in microglial activation, neurogenesis, and BBB permeability.

Scientific evidence indicates that intestinal dysbiosis causes a neuroinflammatory cascade, changes in membrane permeability, in addition to worsening β-amyloid protein aggregation and tau protein hyperphosphorylation.

This article aimed at a narrative review of new knowledge and more recent evidence on the relationship between the brain-microbiota axis and the neuropsychiatric symptoms of AD. Greater efforts should be made to obtain scientific data for the development of therapeutic interventions consisting of the modulation of the intestinal microbiota that provide satisfactory clinical results and an improvement in the quality of life of patients with AD.

## Author Contributions

SQ, AT, and BC: manuscript concept and initial draft. LM and AP: figures and bibliographic research. TP, EV, and MC-T: final version. EV and MC-T: funding and coordination. All authors contributed to the article and approved the submitted version.

## Funding

The authors gratefully acknowledge the State Agency for Development of Science and Innovation of Espírito Santo (FAPES), The National Council for the Development of Science and Technology (CNPq), Agencia Estatal de Investigación. Ministerio de Ciencia e Innovación (Spain) and Xunta de Galicia (Spain), for their contribution to this scientific production with the following Grants: (a) PRONEX (FAPES/CNPq), EV, Edital 24/2018, Termo Outorga 569/2018; (b) PPSUS (FAPES/CNPq/Decit-MS/SESA), EV, Edital 03/2018; Termo Outorga 225/2018; (c) Universal (FAPES), BC, Edital 21/2018, Termo Outorga 120/2019; (d) Agencia Estatal de Investigación, MC-T, (PID2020-119178GB-I00); (e) Xunta de Galicia: Plan Galego IDT, 2021-2022 (Grant Number Code: ED431B 2020/26. Research group GPC GI-18629; (f) Grant FAPES, BC: Processo 552/2018; (g) Grant CNPq, EV: Processo 305740/2019-9; (h) Grant CNPq, TP: Processo 309277/2019-1.

## Conflict of Interest

The authors declare that the research was conducted in the absence of any commercial or financial relationships that could be construed as a potential conflict of interest.

## Publisher's Note

All claims expressed in this article are solely those of the authors and do not necessarily represent those of their affiliated organizations, or those of the publisher, the editors and the reviewers. Any product that may be evaluated in this article, or claim that may be made by its manufacturer, is not guaranteed or endorsed by the publisher.
